# Molecular Advances Leading to Treatment Implications for Fragile X Premutation Carriers

**DOI:** 10.4172/2168-975X.1000119

**Published:** 2014-04-05

**Authors:** Jonathan Polussa, Andrea Schneider, Randi Hagerman

**Affiliations:** 1 Medical Investigation of Neurodevelopmental Disorders (MIND) Institute, University of California Davis Health System, Sacramento, California, USA; 2 Department of Pediatrics, University of California Davis Health System, Sacramento, California, USA

**Keywords:** Fragile X premutation, FXTAS, Aging, Treatment, FMRP, *FMR1*, Antioxidants, Oxidative stress

## Abstract

Fragile X syndrome (FXS) is the most common single gene cause of intellectual disability and it is characterized by a CGG expansion of more than 200 repeats in the *FMR1* gene, leading to methylation of the promoter and gene silencing. The fragile X premutation, characterized by a 55 to 200 CGG repeat expansion, causes health problems and developmental difficulties in some, but not all, carriers. The premutation causes primary ovarian insufficiency in approximately 20% of females, psychiatric problems (including depression and/or anxiety) in approximately 50% of carriers and a neurodegenerative disorder, the fragile X-associated tremor ataxia syndrome (FXTAS), in approximately 40% of males and 16% of females later in life. Recent clinical studies in premutation carriers have expanded the health problems that may be seen. Advances in the molecular pathogenesis of the premutation have shown significant mitochondrial dysfunction and oxidative stress in neurons which may be amenable to treatment. Here we review the clinical problems of carriers and treatment recommendations.

## Introduction

Fragile X premutation carriers are identified by an expanded trinucleotide (CGG) expansion in the 5’ untranslated region of the *FMR1* gene. In the past, research mainly focused on the individuals with the full mutation with fragile X syndrome (FXS) and carriers were thought to be unaffected. In the last 20 years, our knowledge of clinical involvement in premutation carriers has expanded to a broad range of neurological, neurocognitive, endocrine and psychiatric problems related to RNA toxicity [1, 2]. This review will focus on disorders related to the premutation and recommendations for treatment. The premutation is common in the general population and approximately 1 in 130 to 250 women and 1 in 250–810 males have the premutation [3–5].

FMRP, the protein produced by the *FMR1* gene, is important in embryonic development, including the differentiation and migration of neurons and glia cells, for regulation of synaptic plasticity throughout life and for adult neurogenesis [6–9]. FMRP is also critical for normal connectivity with an appropriate balance of excitatory (glutamate) and inhibitory (GABA) circuits [10, 11]. In the absence of FMRP there is a deficit of GABA_A_ activity [12] and up-regulation of the metabotropic glutamate receptor 5 (mGluR5) pathway leading to enhanced long term depression (LTD) of synaptic connections [13]. Hays et al. [14] have demonstrated a prolonged neocortical UP (depolarized firing of neurons) state in FXS mouse that is rescued by mGluR5 antagonists. FMRP also regulates presynaptic release of neurotransmitters. When it is absent or deficient, there is enhanced release which leads to problems in detecting subtle changes in synaptic stimulation [15]. Those with the full mutation have little or no FMRP, whereas the levels of FMRP in carriers of the premutation correlates inversely with CGG repeat number [16, 17]. Most carriers have normal levels of FMRP but those with a premutation above 120 can have significant deficits of FMRP [16–21].

The premutation is associated with significant up-regulation (2 to 8 times normal) of the *FMR1* mRNA that correlates directly with CGG repeat number [22]. Elevated *FMR1* mRNA leads to a process of RNA toxicity which is thought to be the main cause of clinical involvement in premutation carriers [23]. The excess *FMR1* mRNA contains the expanded repeats that form hairpin loops which are sticky and sequester proteins that are needed for normal neuronal function (including Sam 68, DROSHA and DGCR8) [23–25]. The elevated *FMR1* mRNA and the sequestered proteins lead to the formation of inclusions in neurons, astrocytes, and peripheral nervous system and tissue including the adrenals, testes, pancreas, heart, and other organs [26–28].

The development of the knock-in premutation mouse has allowed further studies of the neuronal dysregulation that occurs in carriers. The premutation mouse also develops inclusions and neurological symptoms with aging [29]. There is a deficit of GABA inhibition noted in the mice and also in females with the premutation through transcranial magnetic stimulation (TMS) studies [12, 30]. In premutation neuron cultures, the dendritic tree is less complex with fewer synaptic connections [31]. The mitochondria also have slower movement within dendrites and axons [32] and the neurons have enhanced spikes [33] compared to controls.

There is evidence that both mild deficits of FMRP and the RNA toxicity of elevated *FMR1* mRNA can contribute to the phenotype of premutation carriers [1]. Recent studies have shown that FMRP levels may vary in the general population in those that do not have an *FMR1* mutation [34]. Keri and Benedek [35] studied typical individuals and found that the level of FMRP correlates with studies of visual contrast sensitivity and perception, such that those with a higher level of FMRP have better visual perceptual abilities. Wang et al [36] found that the size of cortical structures correlated with FMRP levels in those without a fragile X mutation. Recently, in those with schizophrenia, it has been found that the age of onset and the IQ correlated with the level of FMRP in blood [34, 37]. Fatemi and colleagues [38, 39] have found that various neuropsychiatric disorders including depression, bipolar disorder, autism, and schizophrenia have a deficit of FMRP in the brain.

**Figure 1:**
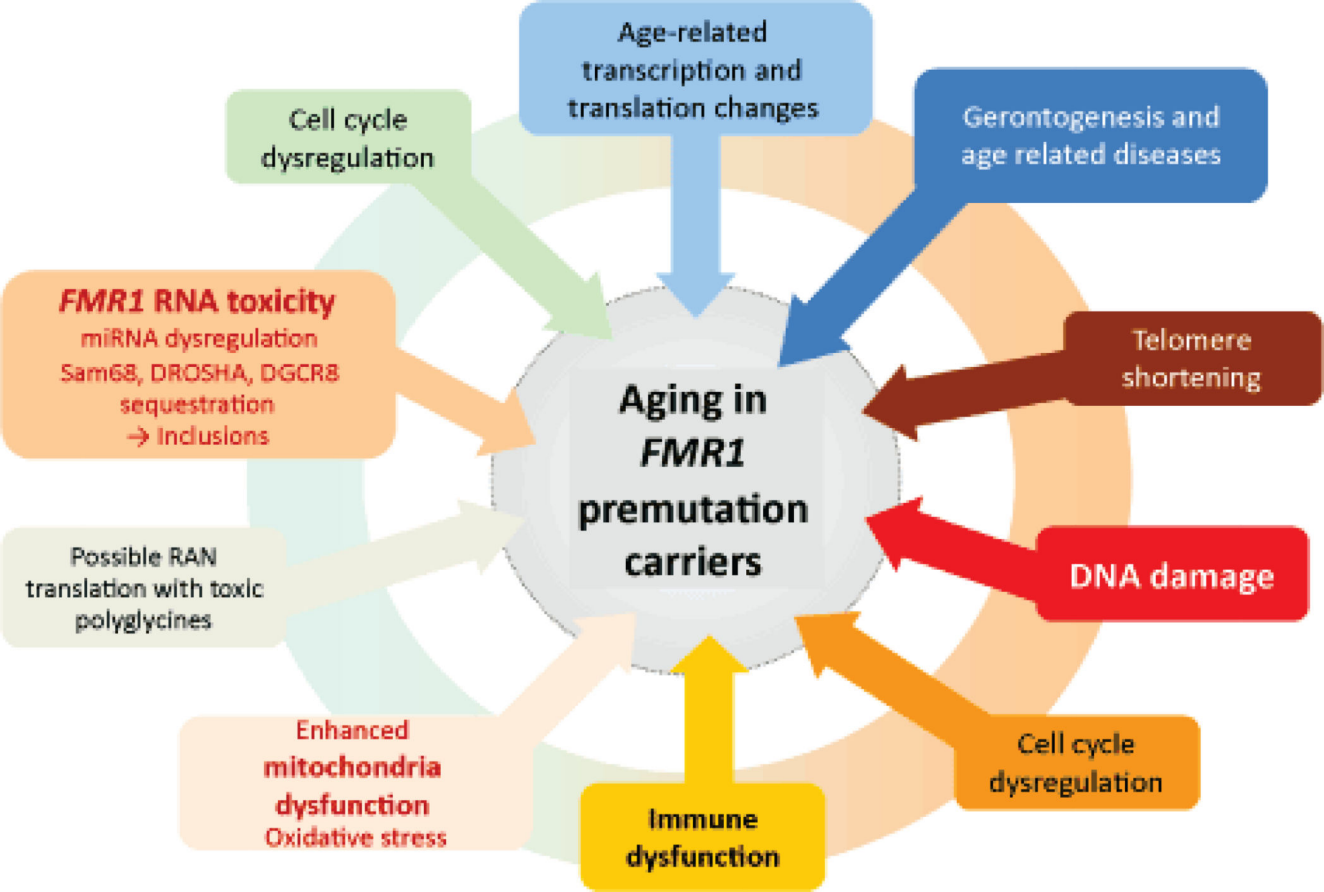
General model of aging in *FMRl* premutation carriers. Normal aging processes, like telomere shortening, gerontogenesis etc., are exacerbated by *FMRl* toxicity across the whole lifespan of a premutation carrier.

Seizures can be deleterious for development and early life seizures in rats without an *FMR1* mutation have been shown to shift FMRP away from the dendritic spines and into the perinuclear area leading to FMRP dysfunction at the synapse [40]. These findings emphasize the need to treat seizures as early as possible so that the levels of FMRP in the dendrites can be sustained. Seizures in boys with the premutation have been associated with the development of autism spectrum disorder (ASD) [41]. Therefore both the molecular findings (CGG repeats, FMRP and *FMR1* mRNA) and environmental experiences such as seizures or exposures to toxins may influence the early development and aging of carriers (Figure 1).

## FXPOI and FXTAS

In 1991 Cronister et al [42] discovered an increased prevalence of premature ovarian failure (POF; menopause before age 40) in females with the premutation that has been confirmed by many others. This condition is now called fragile X-associated primary ovarian insufficiency (FXPOI) [43–45]. FXPOI occurs in approximately 20% of carriers, whereas cessation of periods before age 45 occurs in an additional 20% [46].

Ten years later in 2001, the fragile X-associated tremor ataxia syndrome (FXTAS) was reported in older male carriers [47] followed by a report of FXTAS in females with the premutation [48].

FXTAS is characterized by intention tremor, ataxia leading to frequent falling, peripheral neuropathy, autonomic dysfunction including hypertension, orthostatic hypotension, and cognitive decline [49, 50]. The cognitive problems begin with executive function and short term memory deficits and then gradual cognitive decline occurs, sometimes leading to dementia, particularly in males [51–54]. FXTAS is also characterized by generalized brain atrophy and white matter disease in the periventricular and subcortical regions in addition to the middle cerebellar peduncles (MCP sign) [55]. More recent reports have shown thinning of the corpus callosum with white matter disease in the splenium, pons and insula [1, 56, 57].

The diagnostic criteria for FXTAS were described after the original patients were reported [49]. The original diagnostic guidelines for FXTAS based on clinical reports were later expanded to include characteristic FXTAS eosinophilic intranuclear inclusions that are seen in neurons and astrocytes throughout the brain of individuals with FXTAS [28, 48, 58]. The onset and trajectory of the cognitive problems FXTAS is similar to that of Alzheimer Disease (AD), and, in fact, may benefit from research into treatments for AD [59, 60] or other neurodegenerative diseases such as Parkinson’s disease (PD). Neuropathological studies of those with FXTAS have shown the common co-morbidity of FXTAS, AD and PD suggesting that FXTAS may stimulate the onset of these other aging diseases [1, 59].

## Expanding the Premutation Clinical Picture

We now realize that premutation clinical involvement includes many more conditions than FXTAS or FXPOI alone. In those carriers that have mild deficits of FMRP [20] there may be features of FXS, including prominent ears or more significant deficits in executive function [61, 62] and visuospatial perception, [35, 63],[64, 65]. There are working memory deficits in the premutation carriers both with and without FXTAS [36, 61, 66–68].

Studies of the brain of the premutation mouse models have demonstrated deficits of FMRP throughout the brain [69–71]. The dual mechanism of involvement, of high *FMR1* mRNA and low FMRP is considered a double hit, including features of FXS and premutation involvement. The phenotype of these individuals is somewhat different from typically affected males with FXS because a mild deficit of FMRP usually causes only mild developmental problems so that these individuals have a higher IQ, and less severe behavioral problems than those with full mutation FXS [36, 61, 66–68].

In a national survey study of over 1,276 families who have children with either the full mutation or the premutation, Bailey et al [72] found a high rate of co-morbidity in boys with the premutation. The problems that were significantly different from their non-fragile X brothers included autism in 19.3%, attention problems in 41%, seizures in 11.3%, developmental disabilities in 33%, and anxiety in 33% of the premutation brothers. Similar results were seen by Farzin et al [73] who studied premutation proband brothers who presented clinically compared to premutation brothers who were identified by cascade testing and brothers without the premutation. There was a significantly higher rate of autism, ASD, and ADHD in the probands compared to the other two groups. However, nonproband brothers also had an increase in social deficits compared to the non-premutation brothers. In a subsequent study by Chonchaiya et al [41], boys with the premutation had a higher rate of seizures than controls and the presence of seizures correlated with the presence of ASD. This study suggests that the occurrence of seizures will further interfere with the connectivity of the brain in young carriers and make ASD a more likely outcome. Therefore, early identification and treatment of seizures is a priority in premutation carriers and in those with FXS [74, 75], because seizures can further reduce the available FMRP levels in dendrites [40].

The emotional problems of carriers have been well studied [76–81]. Females with the premutation have been found to have higher rates of mood disorders than the general population, [82] and those with FXTAS have higher rates of major depressive disorder, panic disorder, post-traumatic stress disorder, and specific phobia compared to the general population [83]. Carriers without FXTAS have higher rates of social phobia compared to the general population [83]. These problems are thought to be related to RNA toxicity in the amygdala and hippocampus. MRI imaging has demonstrated that the size of the hippocampus directly correlates with the level of anxiety in females with the premutation who have not developed FXTAS [84]. Therefore, the higher the anxiety level, the smaller the hippocampus suggesting that long term stress and anxiety in carriers is deleterious to the brain. In female carriers, there is a correlation of CGG repeats, cortisol level, negative life events and the risk for the development of emotional problems [2, 81].

Additional medical problems that are common in carriers, particularly aging carriers with neurological problems or FXTAS, include hypothyroidism (50% of females) [85], fibromyalgia (43% of females) [85], sleep apnea [86], migraine headaches [87], Restless Legs syndrome [88], fatigue [89] and hypertension [90]. Regular screening for hypothyroidism, hypertension, migraines, and sleep apnea are recommended because these problems also require treatment and can interfere with brain function if untreated. Olfaction deficits with FXTAS can also cause quality of life issues for premutation carriers [91].

Sleep apnea may often start before the onset of other neurological problems with FXTAS [86]. Therefore, we recommend that all individuals with FXTAS or neurological symptoms be tested for sleep apnea, since the hypoxia generated by sleep apnea may further exacerbate the MRI abnormalities seen in carriers including the white matter disease. Hypertension is significantly increased in FXTAS compared to controls and this may also be exacerbated by sleep apnea. Treatment of hypertension is also a necessity in premutation carriers because uncontrolled hypertension can also lead to further CNS dysfunction including progression of white matter disease and brain atrophy [86, 92].

## MRI studies of Premutation Carriers

Work by Hessl and Rivera has advanced our understanding of the molecular underpinnings of CNS dysfunction causing problems in carriers who do not have FXTAS. In their study of 23 young adult male carriers (mean age 32.9 years), compared to age-matched controls, they found a significantly smaller left and right amygdala volume and a significant decrease in the right amygdala activation in an emotion matching task [93]. The molecular parameters of lowered FMRP levels and the elevated *FMR1* mRNA levels correlated significantly with the decreased activation of the amygdala, but FMRP was the most significant molecular factor in these correlations [93]. Additional MRI studies by Hashimoto et al have shown significant grey matter reduction in the anterior subregions of the cerebellar vermis and hemispheres [94]. Additionally, in carriers without FXTAS, as compared to age-matched controls, display decreased activation in the right ventral inferior frontal cortex and in the left premotor dorsal inferior frontal cortex on fMRI [95]. The problems in the frontal cortex may relate to the executive function and memory deficits seen in some premutation carriers who do not have FXTAS [61, 95]. On DTI studies, there are significant elevations in the axial diffusivity and in the radial diffusivity in the MCP area in carriers without FXTAS compared to age-matched controls [96] in addition to some connectivity loss [97]. A study by Apartis et al. describes corpus callosum splenium hyperintensities as being as frequent as the MCP hyperintensities, especially in female carriers [50].

These changes in the MRI studies on brain structure and function in carriers compared to controls are present in young adults and therefore likely represent long term effects of RNA toxicity in addition to mild deficits of FMRP. It is uncertain which, if any, of these deficits will predict the development of FXTAS, which occurs in 40 % of male carriers and up to 16.5% of female carriers [77, 78, 85, 98]. Typically, the onset of FXTAS occurs in the early 60s, with tremor and then ataxia [54], but, on occasion, FXTAS may present with cognitive decline initially [54, 99]. However, when FXTAS emerges, the abnormalities in white matter are apparent on a clinical reading of the MRI. Battistella et al [100] have noted significant decreases in gray matter in the cerebellar and hippocampal areas of asymptomatic premutation carriers as young as twenty years old (average age of 47) and also that a later onset of diffuse white matter disease may serve as a marker for imminent FXTAS onset [100]. These signs indicate a long progression towards FXTAS and offer hope for a wider window during which treatments may be effective [36, 101].

## Premutation Neurons in Culture

**Figure 2:**
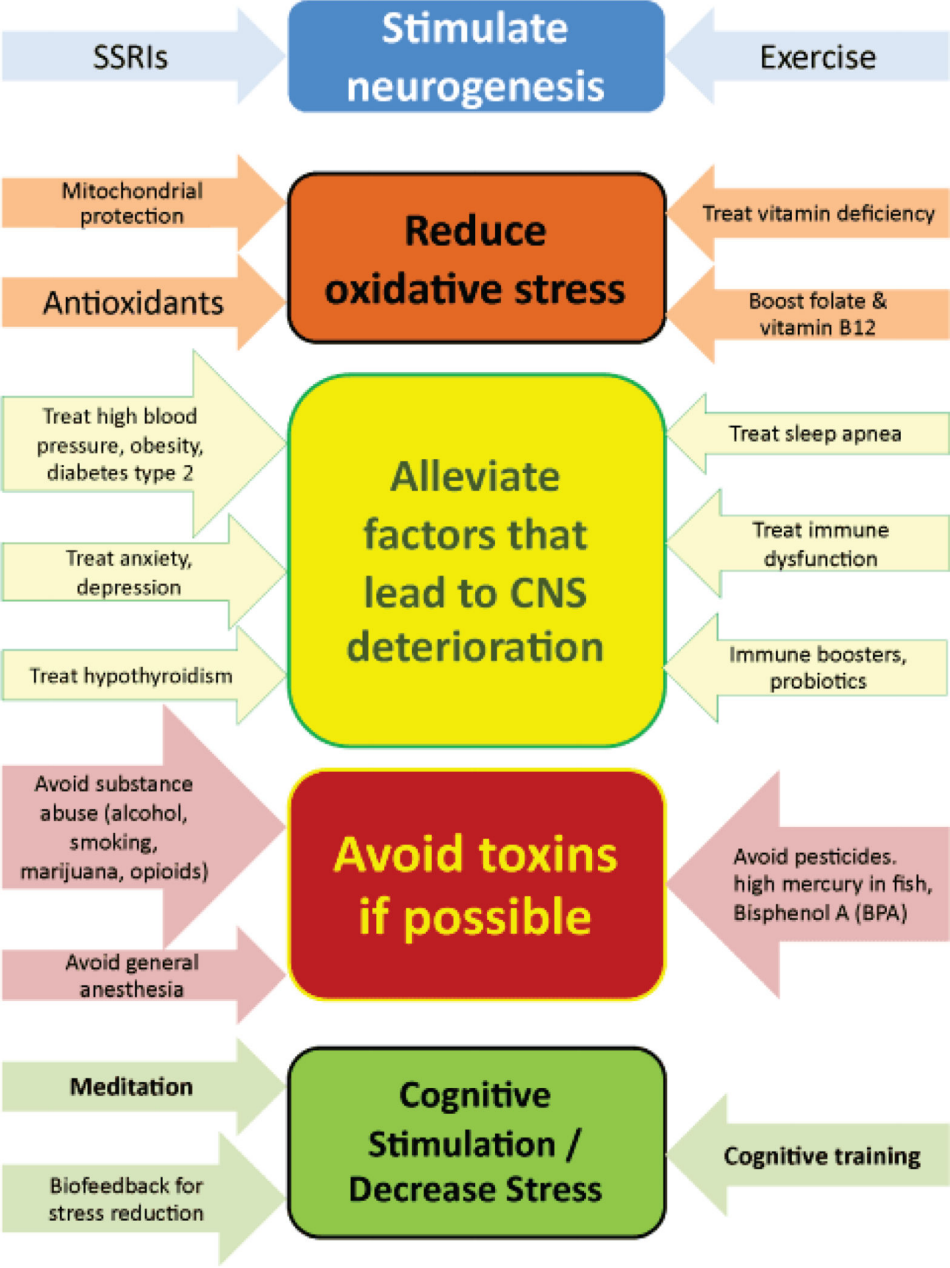
Recommendations and treatments to support healthy aging and support for *FMRl* premutation carriers including medications, complementary and alternative treatment methods, and lifestyle changes as evidenced by similar neurodegenerative disorders.

Chen et al. [31] have studied premutation mouse neurons in culture compared to neurons without the premutation. The complexity of the dendritic tree was reduced and stress proteins were increased in the premutation neurons compared to controls. The premutation neurons would die more easily in culture with a significant increase in the death rate by 21 divisions. Additional morphological differences include reduced postsynaptic density protein 95 (PSD 95) expression, reduced synaptic puncta density, and reduced neurite length [102]. Premutation neurons are also functionally abnormal as clustered spontaneous calcium oscillations have been observed with amplitudes increased over normal [102, 103]. Premutation cells in culture are hyper-responsive to glutamate in that intracellular calcium concentrations remained elevated after exposure [102]. These findings suggest that premutation neurons are more vulnerable to toxicity or stressful conditions in culture, and this has been seen in clinical cases who have enhanced neurological problems when exposed to toxins such as chemotherapy [104] or environmental toxins [105]. Smoking is another toxin that can negatively impact premutation cells. Smoking in females with the premutation can significantly lower the age of FXPOI compared to non-smokers [106]. The premutation neurons demonstrate enhanced oxidative stress and a rise in heat shock proteins (including α B crystallin and dysregulation of lamin A/C) [31, 107]. These findings suggest that treatment with antioxidants would help premutation neurons and cellular studies are underway to assess this effect. Because of the known oxidative stress, we recommend treatment with antioxidants in carriers as described below (Figure 2).

## Mitochondrial Dysfunction and *FMR1* RNA Toxicity

The toxicity that excessive *FMR1* mRNA levels cause in premutation cells may be related to several factors. The dysregulation of lamin A/C is significant, and distortions of nuclear structure can be seen in fibroblasts, neurons and even embryonic cells [107, 108]. The binding and sequestration of DROSHA and DGCR8 can lead to microRNA (miRNA) dysregulation [24]. These two proteins are critical for miRNA maturation, and when they are depleted, related to sequestration, there is dysregulation of the miRNA levels, which are important control elements for most of the activities of the cell. Dysregulation of several miRNA levels have been found those with FXTAS [25, 109]. Additionally, in the Drosophila model of FXTAS, the activation of specific retrotransposons can modulate neurodegeneration [110]. The intrinsic mis-folding and aggregation tendencies of *FMR1* RNA has also been suggested as a possible mechanism in the pathology of the neurodegeneration associated with FXTAS [111]. A recent report of RAN translation in those with FXTAS can lead to the development of toxic levels of polyglycine [112]. Premutation hippocampal neurons have reduced mobility and higher rates of basal oxygen consumption and proton leakage in mouse models [32].

There are also abnormalities of mitochondrial functions related to transport problems of 3 nuclear encoded proteins into the mitochondria in carriers both with and without FXTAS [113, 114]. The brain’s dependence on oxidative phosphorylation of glucose for ATP makes this especially important in neural tissues. In addition to iron dysregulation and deposition, defective zinc bioavailability and/or transport may be related to this mitochondrial dysfunction [114]. This problem can be compounded with environmental stress and/or toxins leading to even more impairment [114]. This is especially problematic as stress mRNAs appear to accumulate in the nucleus of cells, and may be linked to neurodegeneration [115]. Deficits in mitochondrial function are also seen in autism [116] and in other neurodevelopmental disorders in addition to some neurodegenerative disorders including PD and AD [117, 118].

The premutation has been seen in a limited number of other neurodegenerative diseases such as PD [56]. In fact, some cases of PD are nearly indistinguishable from cases of FXTAS [119]. The use of L-DOPA, deep brain stimulation and other treatments for PD may prove helpful for carriers [60, 120].

## FXTAS in Gray Zone and in Unmethylated Full Mutation Carriers

The original diagnostic criteria for FXTAS state that the premutation is necessary for the diagnosis [49]. However, recent reports have documented FXTAS in individuals with a gray zone CGG repeat expansion (45–54 CGG repeats) [121, 122]. In the Liu et al paper, grey zone individuals with tremor and balance problems were in families known to have individuals with FXTAS related to the premutation. Since FXTAS can cluster in families, there may be additional genetic predisposing or protective factors. One report has found that an ApoE4 allele, which predisposes to AD, is also associated with FXTAS [123]. Loesch et al [124] have demonstrated that the gray zone shows elevated *FMR1* mRNA compared to the general population. Thus, it is likely that the elevation in *FMR1* mRNA is sufficient to induce FXTAS through *FMR1* mRNA toxicity. However, since the gray zone is very common in the general population (approximately 1 in 30) most individuals with a gray zone do not develop FXTAS.

Those individuals with an unmethylated full mutation can also develop FXTAS because they have elevated *FMR1* mRNA. The first case was first reported by Loesch et al. [125] and this patient was affected by substance abuse (alcoholism). This patient met all of the criteria of FXTAS including the MCP sign and cognitive decline. It is possible that his alcoholism also predisposed him to FXTAS. We have also seen an unmethylated mosaic patient who presented with a neurodegenerative condition that was diagnosed as PD [121] but, on autopsy, FXTAS inclusions were seen, and he demonstrated the clinical features of FXTAS [126]. Even though these cases may be rare, they suggest that the definition of FXTAS should be extended to include those with a gray zone and an unmethylated full mutation.

## Toxins and Anesthesia

The issue of exacerbation of the molecular dysregulation in fragile X premutation carriers by environmental toxins requires further research [127]. Work in autism has implicated a broad group of toxins including insecticides, pesticides, polychlorinated biphenyls (PCBs), fire retardants, mercury and others [127–132]. Organophosphate pesticides can interfere with acetylcholine and GABA neurotransmission. PCBs and related non-coplanar structures such as triclosan, a common antibacterial, can also activate the ryanodine receptor and mobilize Ca+2, which disrupts signaling and effects both neurodevelopment and neurodegeneration [128]. Recent work by Wolstenholme et al [133] has demonstrated transgenerational disruption of mRNA levels in the brain leading to social deficits related to bisphenol A exposure in mice. These toxins are likely to exacerbate the clinical picture in both premutation carriers and full mutation patients with FXS.

Anesthesia and surgery can also lead to neurotoxicity in the general population, particularly for children who have had multiple surgeries [134, 135] and for more vulnerable populations such as those with AD [136]. In AD, biomarkers for neuroinflammation are increased in cerebrospinal fluid after anesthesia [136]. Amyloid β production and tau phosphorylation are increased as well [136]. Premutation carriers, particularly those with FXTAS, are also a vulnerable population, and we have seen the onset of FXTAS symptoms following a prolonged surgery, particularly in those who are 60 years old and older [1]. However, these cases are anecdotal. There is a need for large-scale studies to understand the link between general anesthesia and the development of FXTAS. Even so, avoidance of prolonged general anesthesia, when possible, is recommended for older carriers, particularly if neurological symptoms or FXTAS are present.

Drugs of abuse are another source of toxins. In our experience, premutation carriers who abuse drugs often have faster progression of their FXTAS symptoms. Heroin, cocaine, methamphetamine, and marijuana induce oxidative stress in neurons [137–143]. The use of drugs and/or excessive alcohol is common in carriers [144, 145], perhaps in an effort to selfmedicate and improve ADHD symptoms, anxiety, and chronic pain caused by neuropathy even prior to FXTAS. However, there may be a biological drive related to elevation of mGluR5 that modulates alcohol self-administration and relapse behavior, potentially allowing for treatment with acamprosate [146, 147].

Animal studies suggest that the oxidative stress related to drugs of abuse can be reversed by antioxidants [137, 148]. Methamphetamine abuse leads to very significant neurotoxicity relating to a long lasting glial response with an increase in the number of astrocytes, release of inflammatory mediators, production of reactive oxygen species (ROS), and an increase in the blood brain barrier permeability through modification of the tight junctions [138]. Methamphetamine abuse augments dopamine levels and dopamine removal by auto-oxidation or monoamine oxidase, leads to the production of ROS. Some of these changes can be improved by antioxidants as described below [137, 139, 148].

Opioids are also concerning because they can lead to exacerbation of white matter disease, yet many premutation carriers are prescribed these drugs because of pain symptoms related to neuropathy or fibromyalgia. In animal models, opioid use has been shown to cause brain damage across a broad range of doses [149] and induces cell death through mitochondrial dysfunction [150]. Similar compounds that act on opioid receptors, such as methadone, should also be avoided due to similar effects [151, 152].

Alcohol is especially important to avoid in excess. Chronic excessive alcohol consumption can cause neuroinflammation, brain damage, and disrupts myelination [153, 154]. Alcohol consumption also decreases white matter integrity [155], potentially complementing a decline into FXTAS as seen in the Loesch et al case [125]. Although red wine, in moderation may protect against neurodegeneration (due to the neuroprotective effects of resveratrol [156], excessive alcohol consumption should be avoided.

## Treatment Implications for Premutation Carriers

A significant question that arises on a regular basis in clinic is what can be done to reduce the risk of developmental problems and aging problems in individuals with the premutation. While longitudinal studies have not yet been done to investigate this issue, current molecular finding (including enhanced molecular stress and mitochondrial dysfunction as described above) suggest the use of treatments proven in similar conditions may be beneficial. Additionally, premutation carriers may be proactive about treating other quality of life aspects of global health impacted by the premutation (such as anxiety or stress) (Figure 2), especially as it has been suggested that there exists a limited developmental window during which intervention would be most effective [157].

## Medical Interventions

Although no medication has been found to block or reverse FXTAS so far, new research has opened possibilities of medical interventions to help mitigate some of the problems with the premutation throughout the lifespan. Interventions are being studied now to lower the excess *FMR1* mRNA, but they are not available clinically yet [23]. A controlled trial of memantine to block glutamate toxicity exacerbated by glutamate abnormalities in carriers [103] was not shown to be efficacious for the tremor, balance or executive function deficits in FXTAS [158]. However, ERP studies have shown improvement in brain-language processing in those with FXTAS treated with memantine compared to controls, so there may be some minimal cognitive improvements in processing information [159]. Allopregnanolone, a natural neurosteroid, has shown some benefit to the mouse model of premutation. When administered to premutation neurons in culture, allopregnanolone mitigated functional impairments observed in premutation neurons in a reversible manner [33]. The use of an mGluR5 antagonist (MPEP) was also helpful to the premutation neurons, suggesting that this category of targeted treatments for FXS may be helpful in FXTAS.

When depression or anxiety are present, selective serotonin reuptake inhibitors (SSRIs) are recommended, and their effect is usually beneficial [160]. SSRIs not only increase serotonin levels, but they also stimulate neurogenesis [161–163] through increased levels of BDNF [164]. This may be helpful in FXTAS individuals who have significant brain atrophy [60]. Encouraging neurogenesis may facilitate prevention and treatment of depression as well [162, 163].

### Antioxidants

Based on animal studies or human studies on disorders similar to the premutation, there are a variety of antioxidants that are likely to be helpful in reversing the oxidative stress of neurons with the premutation (Table 1). In newborn KO mice, de Diego-Otero et al demonstrated that treatment with alpha-tocopherol (vitamin E), in addition to N acetyl –L cysteine (NAC), normalized synaptic connections and rescued aspects of the physical and cognitive/ behavioral phenotype [165]. Additional studies have linked vitamin E to improvements in dementia associated with AD as well as generalized mild cognitive impairments common with age [166, 167]. Chronic pharmacological treatment with alphatocopherol (Vitamin E) in fragile X mice has been shown to alleviate free radical overproduction, and reduce oxidative stress [165]. The same study also showed improved behavior and reversed learning deficits [165]. Vitamin E and other vitamins are likely helpful for premutation carriers as well. However, further studies of these antioxidants have not been carried out in patients with the premutation.

Melatonin is another antioxidant where use in *FMR1* knockout mice normalized glutathione levels compared to controls [168]. Melatonin has also shown to improve context-depended exploratory and anxiety behaviors and learning abnormalities [168]. Melatonin has also shown to be an effective sleep aid in individuals with FXS [169]. As sleep disturbances are very common in individuals with the premutation, particularly in carrier daughters of men who have FXTAS [170], earlier and longer sleep may significantly improve quality of life for premutation carriers. Melatonin, while effective for sleep, is also effective in preventing oxidative stress in models of PD and AD [171]. Melatonin is also unique in that it is selectively taken up by mitochondrial membranes [171] which may enhance its protective action in the premutation.

Folic acid is an antioxidant that has been studied for years in individuals with FXS [173, 174]. It has a minimal psychotropic effect in some controlled studies, although the reason for this effect is not known. It is clearly important for aging because folic acid, combined with vitamin B12, lowers homocysteine levels. This combination reduces brain atrophy in typically aging individuals and also in those with mild cognitive impairment (MCI) [175]. Since brain atrophy is a severe problem in older premutation carriers with FXTAS, we routinely recommend treatment with folic acid and vitamin B 12 in older carriers to lower homocysteine levels.

Coenzyme Q10 (CoQ10) acts as an electron carrier in the electron transport chain of mitochondria and appears to have antioxidant effects. CoQ10 deficiencies have been documented in diseases similar to FXTAS such as PD and AD [178, 193]. Although more studies are needed, widespread preliminary data shows that CoQ10 have shown encouraging data results in treating neurological disorders such as Alzheimer, Parkinson, Friedrich and Huntington diseases [176–180, 194].

Other antioxidants that may prove beneficial to premutation carriers include ginseng and ginsenosides. Korean Red Ginseng has been shown to protect mitochondria from damage, prevent cell death during iron-induced oxidative stress, [181] and also to prevent neuroinflammation associated with neurodegeneration [182]. In mice, ginseng shows a fluoxetine-like anti-depressant effect and regulates synaptic plasticity proteins (including BDNF) to prevent cognitive decline associated with aging [183, 184].

Omega 3 fatty acids also have a protective function against oxidative stress. Human studies show reduced oxidative stress and damage [185]. Omega 3s increase superoxide dismutase activity in rats [186] where it is also shown that induced oxidative stress is compensated for through daily doses of omega 3s [187].

Anecdotally, patients with FXTAS have commented on beneficial effects of L-Arginine. There are some studies in rats that support a protective effect of L-Arginine against oxidative stress [195], however this, in addition to other antioxidants, have not been studied in premutation carriers.

Glutathione is also an essential cofactor for glutathione peroxidase, which acts as an antioxidant enzyme in the cell. Deficits of glutathione peroxidase cause additional oxidative stress in cells. Anthocyanin class compounds are effective in reducing the iron based lipid peroxidation and overall levels of oxidative stress in cells [192]. Additionally, anthocyanins significantly enhance glutathione peroxidase activity in cell-free assays [192].

Epigallocatechin-3-gallate (EGCG), found in green tea, is another potent antioxidant whose antioxidant effects in-vivo are well known [188–190, 196]. Consumption of green tea has been shown to alleviate age-related neuro-degeneration and boost cognitive function and would thus likely be helpful for FXTAS [189, 191, 196].

A study by Bowman et al [197] analyzed plasma biomarkers as a measure of nutritional quality. Higher levels of plasma vitamins and marine omega 3 fatty acids were linked to higher cognitive ability and higher brain volume with aging. Conversely, the study found that higher levels of trans-fats were linked to lower cognitive ability and reduced brain volume [197]. This evidence suggests that such changes in diet may be helpful for FXTAS with aging also.

While each of these vitamins and antioxidants have shown significant protection against oxidative stress, none have been studied specifically in premutation carriers with or without FXTAS. There is a need for such studies. Although these interventions are not likely to reverse FXTAS, they may be important, along with other lifestyle changes, to stall, delay, or avoid the onset of FXTAS in carriers.

### Stress reduction

Raising a child with FXS, taking care of someone with FXTAS, or other significant involvement from the premutation is stressful to parents and to the family [80, 198]. Animal models of the premutation have also shown increased stress and cortisol elevations with age [199]. Mindfulness based stress reduction has been studied for reducing stress in cases of long term conditions with psychological distress and well-being issues such as cancer [200], multiple sclerosis [201], fibromyalgia [202] and rheumatoid arthritis [203] and have yielded positive results. Fibromyalgia is common in female premutation female carriers with age and the prevalence rises to 43% if neurological problems/FXTAS are present [85, 204]. Mindfulness stress reduction is a mental training to develop awareness and acceptance skills to cope with daily events that otherwise cause heightened elevated stress. Mindfulness meditation has been shown to reduce anxiety, improve mood, and increase brain GABA levels [205].

Relaxation is a process that decreases the effects of stress and supports stress management. Beneficial effects of relaxation techniques include: slowing heart and breathing rate, lowering blood pressure, increased blood flow, reduced muscle tension and pain, and improved concentration. The National Center for Complementary and Alternative Medicine (NCCAM) published an overview about relaxation techniques for health [206].

Bio and neurofeedback treatments for relaxation and other behavioral interventions have been reported on for over 30 years [207]. Biofeedback and neurofeedback provides specific information about internal biological processes (i.e. muscle activity, respiration, heart rate variability, skin temperature and brain electrical activity) in an individual. Depending upon the physiological processes targeted, healthier patterns of activity can be achieved by most people after they have participated in 10 to 50 sessions of biofeedback or neurofeedback supported with professional coaching and practice. For example, Thompson, Thompson and Reid [208] described that effective neurofeedback plus biofeedback for individuals with Asperger disorder included several 50-minute sessions of neurofeedback twice weekly. Ten to fifteen of these sessions combined neurofeedback with biofeedback of other physiological parameters, such as heart rate variability, respiration, or electrodermal activity, if an initial assessment suggested that the individual experienced difficulties sustaining alertness or heightened anxiety. The therapists also guided individuals to practice and generalize these skills to daily activities. Further, once the individual achieved a relaxed, calm and focused state during the feedback training, they were also trained to maintain this state while performing mental activities designed to improve their cognitive processes (e.g., emotional comprehension in listening).

The 2011 study by Prinsloo et al. [209] showed an improvement in cognitive performance in healthy male volunteers (aged 23 to 41 years old) after the use of a short duration heart rate variability (HRV) biofeedback intervention with the StressEraser, a handheld portable HRV biofeedback device (StressEraser TM, Helicor, USA). Various biofeedback protocols [210] and assistive electronic technologies such as the NeXus-10, emWave Personal Stress Reliever® or StressEraser® exist to enhance the balance of parasympathetic activity, vagal tone, increase HRV and synchronize respiration with the heart rhythm (i.e., the slowing down and speeding up of the heart over time). The emWave® Coherence System by HeartMath is one example that has an emerging evidence base for its efficacy with children [211, 212]. Although these techniques have not been studied in premutation carriers with or without FXTAS, the need is great, and they are worthy of future investigations.

### Exercise, Cognitive training, and Neurogenesis

To complement the use of antioxidants to protect against oxidative stress, exercise may also prove beneficial. Among regularly exercising older men, there is a marked decrease in vascular endothelial oxidative stress [213]. This likely holds true for brain tissue as well. A separate study by Shanely et al finds overall lower levels of inflammation and oxidative stress in adults who are physically fit and regularly active [214, 215]. Furthermore, Traustadottir et al have found that exercise helps to blunt the body’s hypothalamicpituitary-adrenal (HPA) axis response to psychological stress [216].

Exercise offers other benefits as well. Physical exercise increases adult neurogenesis and telomerase activity [161, 217, 218]. Telomeres are shorter in carriers compared to controls, which exercise would likely improve [219]. Exercise in rats boosts immune and neuroimmune cytokines [218]. Exercise also increases the size and plasticity of the hippocampus and improves memory [220, 221]. Although exercise increases the levels of brain-derived neurotrophic factor (BDNF) it appears that the increase in hippocampus size is independent of BDNF levels [215, 221]. Exercise in the mouse model of schizophrenia improves behavioral deficits [217]. Exercise also reduces feelings of depression perhaps by increasing serotonin levels [222]. A daily regimen of resistance training can also increase general cognition [223] and produces a shift in mitochondrial DNA that dilutes mutational burdens associated with aging [224]. Even brief bouts of activity increased levels of mitochondria in cells and helps cope with stress [225].

Several studies have shown beneficial effects of cognitive training to improve cognitive abilities [226, 227]. Cognitive training can be computer-based, and it typically involves a guided practice of standard tasks to reflect areas of cognition, such as memory, attention, or executive function.

## Conclusions

The premutation is common in the general population and it can be associated with both developmental problems in addition to neurological and psychiatric problems with aging. The molecular underpinnings of premutation involvement are known and treatment for specific difficulties including ASD, anxiety, and depression often responds well to the use of an SSRI and counseling/ therapy. Prophylactic intervention with antioxidants, exercise, mindfulness meditation and avoidance of toxins has been anecdotally helpful, although controlled studies are needed to judge efficacy. This issue is complex because it is unclear why some individuals with the premutation have developmental and aging problems, whereas others do not. The reasons are likely related to both environmental and genetic effects. Treatments outlined here may offer some benefit for premutation carriers.

## Figures and Tables

**Table 1:** Antioxidant for fragile X premutation carriers as evidenced by their use in similar population.

Substance	Outcome	Study Population	Reference
Alpha Tocopherol (Vitamin E)	Normalization of synaptic connections	*FMR1* KO Mouse	[165]
	Improvements in dementia	Alzheimer disease	[166, 167]
	Reduction of oxidative stress	*FMR1* KO Mouse	[165]
Melatonin	Glutathione level normalization	*FMR1* KO Mouse	[168]
	Improvement of anxiety and learning abnormalities	*FMR1* KO Mouse	[168]
	Sleep Aid	Children with fragile X	[169]
		Women with the premutation	[170]
	Reduction of oxidative stress	Parkinson disease	[171, 172]
Folic Acid (Vitamin B9)	Lowers homocysteine levels	Fragile X	[173, 174]
	Reduced brain atrophy with age	Typically aging individuals	[175]
CoEnzyme Q10	Global symptoms (UPDRS Score –Unified Parkinson’s Disease Rating Scale)	Parkinson Disease	[176, 177]
	Cognitive Function	Alzheimer Disease	[178]
	Overall energy level	Friedreich Ataxia	[179]
	Early tremor symptoms	Huntington Disease	[180]
Ginseng	Reduction of oxidative stress	Cell cultures	[181]
	Reduction of neuroinflammation	Mice	[182]
	Mood	Mice	[183]
	Memory	Mice	[184]
Omega 3s	Improved antioxidant activity	Humans	[185]
		Rats	[186, 187]
Epigallocatechin-3-gallate (EGCG)	Reduction of oxidative stress	Cell cultures	[188–190]
		Humans	[189]
		Rats	[191]
Anthocyanins	Reduction of oxidative stress	Cell cultures	[192]
N acetyl–L cysteine	Normalization of synaptic connections	*FMR1* KO Mouse	[165]

## References

[1] Hagerman R, Hagerman P (2013). Advances in clinical and molecular understanding of the FMR1 premutation and fragile X-associated tremor/ataxia syndrome. Lancet Neurol.

[2] Kraan CM (2013). Neurobehavioural evidence for the involvement of the FMR1 gene in female carriers of fragile X syndrome. Neurosci Biobehav Rev.

[3] Fernandez-Carvajal I (2009). Screening for expanded alleles of the FMR1 gene in blood spots from newborn males in a Spanish population. J Mol Diagn.

[4] Hagerman PJ (2008). The fragile X prevalence paradox. J Med Genet.

[5] Tassone F (2012). FMR1 CGG allele size and prevalence ascertained through newborn screening in the United States. Genome Med.

[6] Till SM (2010). The developmental roles of FMRP. Biochem Soc Trans.

[7] Castren M (2005). Altered differentiation of neural stem cells in fragile X syndrome. Proc Natl Acad Sci U S A.

[8] Tervonen TA (2009). Aberrant differentiation of glutamatergic cells in neocortex of mouse model for fragile X syndrome. Neurobiol Dis.

[9] Luo Y (2010). Fragile x mental retardation protein regulates proliferation and differentiation of adult neural stem/progenitor cells. PLoS Genet.

[10] Bureau I, Shepherd GM, Svoboda K (2008). Circuit and plasticity defects in the developing somatosensory cortex of FMR1 knock-out mice. J Neurosci.

[11] Gibson JR (2008). Imbalance of neocortical excitation and inhibition and altered UP states reflect network hyperexcitability in the mouse model of fragile X syndrome. J Neurophysiol.

[12] D'Hulst C (2009). Expression of the GABAergic system in animal models for fragile X syndrome and fragile X associated tremor/ataxia syndrome (FXTAS). Brain Res.

[13] Dolen G (2010). Mechanism-based approaches to treating fragile X. Pharmacol Ther.

[14] Hays SA, Huber KM, Gibson JR (2011). Altered neocortical rhythmic activity states in Fmr1 KO mice are due to enhanced mGluR5 signaling and involve changes in excitatory circuitry. J Neurosci.

[15] Deng PY (2013). FMRP regulates neurotransmitter release and synaptic information transmission by modulating action potential duration via BK channels. Neuron.

[16] Pretto DI (2014). CGG allele size somatic mosaicism and methylation in FMR1 premutation alleles. J Med Genet.

[17] Ludwig AL (2014). CNS expression of murine fragile X protein (FMRP) as a function of CGG-repeat size. Hum Mol Genet.

[18] Tassone F (2000). Transcription of the FMR1 gene in individuals with fragile X syndrome. Am J Med Genet.

[19] Kenneson A (2001). Reduced FMRP and increased FMR1 transcription is proportionally associated with CGG repeat number in intermediate-length and premutation carriers. Hum Mol Genet.

[20] Tassone F (2000). Clinical involvement and protein expression in individuals with the FMR1 premutation. Am J Med Genet.

[21] Loesch DZ, Huggins RM, Hagerman RJ (2004). Phenotypic variation and FMRP levels in fragile X. Ment Retard Dev Disabil Res Rev.

[22] Tassone F (2000). Elevated levels of FMR1 mRNA in carrier males: a new mechanism of involvement in the fragile-X syndrome. Am J Hum Genet.

[23] Hagerman P (2013). Fragile X-associated tremor/ataxia syndrome (FXTAS): pathology and mechanisms. Acta Neuropathol.

[24] Sellier C (2013). Sequestration of DROSHA and DGCR8 by expanded CGG RNA repeats alters microRNA processing in fragile X-associated tremor/ataxia syndrome. Cell Rep.

[25] Sellier C (2010). Sam68 sequestration and partial loss of function are associated with splicing alterations in FXTAS patients. EMBO J.

[26] Greco CM (2007). Testicular and pituitary inclusion formation in fragile X associated tremor/ataxia syndrome. J Urol.

[27] Hunsaker MR (2011). Rare intranuclear inclusions in the brains of 3 older adult males with fragile x syndrome: implications for the spectrum of fragile x-associated disorders. J Neuropathol Exp Neurol.

[28] Greco CM (2006). Neuropathology of fragile X-associated tremor/ataxia syndrome (FXTAS). Brain.

[29] Zongaro S (2013). The 3′ UTR of FMR1 mRNA is a target of miR-101, miR-129-5p and miR-221: implications for the molecular pathology of FXTAS at the synapse. Human molecular genetics.

[30] Conde V (2013). Abnormal GABA-mediated and cerebellar inhibition in women with the fragile X premutation. J Neurophysiol.

[31] Chen YC (2010). Murine hippocampal neurons expressing Fmr1 gene premutations show early developmental deficits and late degeneration. Human Molecular Genetics.

[32] Kaplan ES (2012). Early mitochondrial abnormalities in hippocampal neurons cultured from Fmr1 pre-mutation mouse model. J Neurochem.

[33] Cao Z (2012). Clustered burst firing in FMR1 premutation hippocampal neurons: amelioration with allopregnanolone. Hum Mol Genet.

[34] Kelemen O, Kovacs T, Keri S (2013). Contrast, motion, perceptual integration, and neurocognition in schizophrenia: the role of fragile-X related mechanisms. Prog Neuropsychopharmacol Biol Psychiatry.

[35] Keri S, Benedek G (2011). Fragile X protein expression is linked to visual functions in healthy male volunteers. Neuroscience.

[36] Wang JY (2013). Influence of the fragile X mental retardation (FMR1) gene on the brain and working memory in men with normal FMR1 alleles. Neuroimage.

[37] Kovacs T, Kelemen O, Keri S (2013). Decreased fragile X mental retardation protein (FMRP) is associated with lower IQ and earlier illness onset in patients with schizophrenia. Psychiatry Res.

[38] Folsom TD, Fatemi SH (2013). The involvement of Reelin in neurodevelopmental disorders. Neuropharmacology.

[39] Fatemi SH, Folsom TD (2011). The role of fragile X mental retardation protein in major mental disorders. Neuropharmacology.

[40] Bernard PB (2013). Phosphorylation of FMRP and alterations of FMRP complex underlie enhanced mLTD in adult rats triggered by early life seizures. Neurobiol Dis.

[41] Chonchaiya W (2012). Increased prevalence of seizures in boys who were probands with the FMR1 premutation and co-morbid autism spectrum disorder. Hum Genet.

[42] Cronister A (1991). Heterozygous fragile X female: historical, physical, cognitive, and cytogenetic features. Am J Med Genet.

[43] Sullivan AK (2005). Association of FMR1 repeat size with ovarian dysfunction. Hum Reprod.

[44] Wittenberger MD (2007). The FMR1 premutation and reproduction. Fertil Steril.

[45] Welt CK (2008). Primary ovarian insufficiency: a more accurate term for premature ovarian failure. Clin Endocrinol (Oxf).

[46] Sullivan SD, Welt C, Sherman S (2011). FMR1 and the continuum of primary ovarian insufficiency. Semin Reprod Med.

[47] Hagerman RJ (2001). Intention tremor, parkinsonism, and generalized brain atrophy in male carriers of fragile X. Neurology.

[48] Hagerman RJ (2004). Fragile-X-associated tremor/ataxia syndrome (FXTAS) in females with the FMR1 premutation. Am J Hum Genet.

[49] Jacquemont S (2003). Fragile X premutation tremor/ataxia syndrome: molecular, clinical, and neuroimaging correlates. Am J Hum Genet.

[50] Apartis E (2012). FXTAS: new insights and the need for revised diagnostic criteria. Neurology.

[51] Seritan AL (2008). Dementia in fragile X-associated tremor/ataxia syndrome (FXTAS): comparison with Alzheimer's disease. Am J Med Genet B Neuropsychiatr Genet.

[52] Berry-Kravis E (2007). Fragile X-associated tremor/ataxia syndrome: clinical features, genetics, and testing guidelines. Mov Disord.

[53] Jacquemont S (2007). Fragile-X syndrome and fragile X-associated tremor/ataxia syndrome: two faces of FMR1. Lancet Neurol.

[54] Leehey MA (2007). Progression of tremor and ataxia in male carriers of the FMR1 premutation. Mov Disord.

[55] Brunberg J (2003). Fragile X-associated tremor/ataxia syndrome (FXTAS): Autopsy Brain MR Imaging Alterations Correlated with Histopathology.

[56] Loesch DZ (2011). White matter changes in basis pontis in small expansion FMR1 allele carriers with parkinsonism. Am J Med Genet B Neuropsychiatr Genet.

[57] Moris G (2010). Hyperintensity in the basis pontis: atypical neuroradiological findings in a woman with FXTAS. Mov Disord.

[58] Hagerman PJ, Hagerman RJ (2004). The fragile-X premutation: a maturing perspective. Am J Hum Genet.

[59] Tassone F (2012). Neuropathological, clinical and molecular pathology in female fragile X premutation carriers with and without FXTAS. Genes Brain Behav.

[60] Hagerman RJ (2008). Treatment of fragile X-associated tremor ataxia syndrome (FXTAS) and related neurological problems. Clin Interv Aging.

[61] Grigsby J (2008). Cognitive profile of fragile X premutation carriers with and without fragile Xassociated tremor/ataxia syndrome. Neuropsychology.

[62] Loesch DZ (2008). A low symptomatic form of neurodegeneration in younger carriers of the FMR1 premutation, manifesting typical radiological changes. J Med Genet.

[63] Goodrich-Hunsaker NJ (2011). Adult Female Fragile X Premutation Carriers Exhibit Age- and CGG Repeat Length-Related Impairments on an Attentionally Based Enumeration Task. Front Hum Neurosci.

[64] Goodrich-Hunsaker NJ (2011). Young adult female fragile X premutation carriers show age- and genetically-modulated cognitive impairments. Brain Cogn.

[65] Hocking DR, Kogan CS, Cornish KM (2012). Selective spatial processing deficits in an at-risk subgroup of the fragile X premutation. Brain Cogn.

[66] Kraan CM (2013). Cognitive-motor interference during postural control indicates at-risk cerebellar profiles in females with the FMR1 premutation. Behav Brain Res.

[67] Kogan CS, Cornish KM (2010). Mapping self-reports of working memory deficits to executive dysfunction in Fragile X Mental Retardation 1 (FMR1) gene premutation carriers asymptomatic for FXTAS. Brain Cogn.

[68] Fischer A (2007). Recovery of learning and memory is associated with chromatin remodelling. Nature.

[69] Entezam A (2007). Regional FMRP deficits and large repeat expansions into the full mutation range in a new Fragile X premutation mouse model. Gene.

[70] Brouwer JR (2008). CGG-repeat length and neuropathological and molecular correlates in a mouse model for fragile X-associated tremor/ataxia syndrome. J Neurochem.

[71] Qin M (2011). A mouse model of the fragile X premutation: effects on behavior, dendrite morphology, and regional rates of cerebral protein synthesis. Neurobiol Dis.

[72] Bailey DB (2008). Co-occurring conditions associated with FMR1 gene variations: findings from a national parent survey. Am J Med Genet A.

[73] Farzin F (2006). Autism spectrum disorders and attention-deficit/hyperactivity disorder in boys with the fragile X premutation. J Dev Behav Pediatr.

[74] Berry-Kravis E, Knox A, Hervey C (2011). Targeted treatments for fragile X syndrome. J Neurodev Disord.

[75] Garcia-Nonell C (2008). Secondary medical diagnosis in fragile X syndrome with and without autism spectrum disorder. Am J Med Genet A.

[76] Hessl D (2005). Abnormal elevation of FMR1 mRNA is associated with psychological symptoms in individuals with the fragile X premutation. Am J Med Genet B Neuropsychiatr Genet.

[77] Rodriguez-Revenga L (2010). Motor and mental dysfunction in mother-daughter transmitted FXTAS. Neurology.

[78] Rodriguez-Revenga L (2009). Penetrance of FMR1 premutation associated pathologies in fragile X syndrome families. Eur J Hum Genet.

[79] Rodriguez-Revenga L (2008). Evidence of depressive symptoms in fragile-X syndrome premutated females. Psychiatr Genet.

[80] Hartley SL (2012). Cortisol response to behavior problems in FMR1 premutation mothers of adolescents and adults with fragile X syndrome: A diathesis-stress model. Int J Behav Dev.

[81] Seltzer MM (2012). Differential sensitivity to life stress in FMR1 premutation carrier mothers of children with fragile X syndrome. Health Psychol.

[82] Roberts JE (2009). Mood and anxiety disorders in females with the FMR1 premutation. Am J Med Genet B Neuropsychiatr Genet.

[83] Bourgeois JA (2011). Lifetime prevalence of mood and anxiety disorders in fragile X premutation carriers. J Clin Psychiatry.

[84] Adams PE (2010). Psychological symptoms correlate with reduced hippocampal volume in fragile X premutation carriers. Am J Med Genet B Neuropsychiatr Genet.

[85] Coffey SM (2008). Expanded clinical phenotype of women with the FMR1 premutation. Am J Med Genet A.

[86] Hamlin A (2011). Sleep apnea in fragile X premutation carriers with and without FXTAS. Am J Med Genet B Neuropsychiatr Genet.

[87] Au J (2013). Prevalence and risk of migraine headaches in adult fragile X premutation carriers. Clin Genet.

[88] Summers SM (2013). Prevalence of restless legs syndrome and sleep quality in carriers of the fragile X premutation. Clinical Genetics.

[89] Summers SM (2014). Fatigue and body mass index in the Fragile X premutation carrier. Fatigue: Biomedicine, Health & Behavior.

[90] Hamlin AA (2012). Hypertension in FMR1 premutation males with and without fragile X-associated tremor/ataxia syndrome (FXTAS). Am J Med Genet A.

[91] Juncos JL (2012). Olfactory dysfunction in fragile X tremor ataxia syndrome. Mov Disord.

[92] Sierra C, Coca A (2006). White matter lesions and cognitive impairment as silent cerebral disease in hypertension. Scientific World Journal.

[93] Hessl D (2011). Decreased fragile X mental retardation protein expression underlies amygdala dysfunction in carriers of the fragile X premutation. Biol Psychiatry.

[94] Hashimoto R (2011). A voxel-based morphometry study of grey matter loss in fragile X-associated tremor/ataxia syndrome. Brain.

[95] Hashimoto R (2011). An fMRI study of the prefrontal activity during the performance of a working memory task in premutation carriers of the fragile X mental retardation 1 gene with and without fragile X-associated tremor/ataxia syndrome (FXTAS). J Psychiatr Res.

[96] Hashimoto R (2011). Diffusion tensor imaging in male premutation carriers of the fragile X mental retardation gene. Mov Disord.

[97] Wang JY (2012). Age-dependent structural connectivity effects in fragile x premutation. Arch Neurol.

[98] Jacquemont S (2004). Penetrance of the fragile X-associated tremor/ataxia syndrome in a premutation carrier population. JAMA.

[99] Sevin M (2009). Penetrance of marked cognitive impairment in older male carriers of the FMR1 gene premutation. J Med Genet.

[100] Battistella G (2013). Brain structure in asymptomatic FMR1 premutation carriers at risk for fragile X-associated tremor/ataxia syndrome. Neurobiol Aging.

[101] Wang JY (2013). Fragile X-associated tremor/ataxia syndrome: influence of the FMR1 gene on motor fiber tracts in males with normal and premutation alleles. JAMA Neurol.

[102] Liu J (2012). Signaling defects in iPSC-derived fragile X premutation neurons. Hum Mol Genet.

[103] Cao Z (2013). Enhanced asynchronous Ca(2+) oscillations associated with impaired glutamate transport in cortical astrocytes expressing Fmr1 gene premutation expansion. J Biol Chem.

[104] O'Dwyer JP (2005). Fragile X-associated tremor/ataxia syndrome presenting in a woman after chemotherapy. Neurology.

[105] Paul R (2010). Early onset of neurological symptoms in fragile X premutation carriers exposed to neurotoxins. Neurotoxicology.

[106] Allen E (2007). Examination of reproductive aging milestones among women who carry the FMR1 premutation. Human reproduction.

[107] Garcia-Arocena D (2010). Fibroblast phenotype in male carriers of FMR1 premutation alleles. Hum Mol Genet.

[108] Garcia-Arocena D, Hagerman PJ (2010). , Advances in understanding the molecular basis of FXTAS. Hum Mol Genet.

[109] Tan H (2012). MicroRNA-277 modulates the neurodegeneration caused by Fragile X premutation rCGG repeats. PLoS Genet.

[110] Tan H (2012). Retrotransposon activation contributes to fragile X premutation rCGG-mediated neurodegeneration. Hum Mol Genet.

[111] Sjekloca L, Pauwels K, Pastore A (2011). On the aggregation properties of FMRP--a link with the FXTAS syndrome?. FEBS J.

[112] Todd PK (2013). CGG repeat-associated translation mediates neurodegeneration in fragile X tremor ataxia syndrome. Neuron.

[113] Ross-Inta C (2010). Evidence of mitochondrial dysfunction in fragile X-associated tremor/ataxia syndrome. Biochem J.

[114] Napoli E (2011). Altered zinc transport disrupts mitochondrial protein processing/import in fragile X-associated tremor/ataxia syndrome. Hum Mol Genet.

[115] Qurashi A (2011). Nuclear accumulation of stress response mRNAs contributes to the neurodegeneration caused by Fragile X premutation rCGG repeats. PLoS Genet.

[116] Giulivi C (2010). Mitochondrial dysfunction in autism. JAMA.

[117] Schapira AH (2011). Mitochondrial pathology in Parkinson's disease. Mt Sinai J Med.

[118] Du H, Guo L, Yan SS (2012). Synaptic mitochondrial pathology in Alzheimer's disease. Antioxid Redox Signal.

[119] Hall DA (2009). Parkinsonism in FMR1 premutation carriers may be indistinguishable from Parkinson disease. Parkinsonism Relat Disord.

[120] Tassone F, Berry-Kravis E (2011). Fragile X-associated Tremor Ataxia Syndrome (FXTAS).

[121] Hall DA (2011). FMR1 gray-zone alleles: association with Parkinson's disease in women?. Mov Disord.

[122] Liu Y (2013). Fragile X-associated tremor/ataxia syndrome (FXTAS) in grey zone carriers. Clin Genet.

[123] Silva F (2013). High apolipoprotein E4 allele frequency in FXTAS patients. Genet Med.

[124] Loesch DZ (2007). Transcript levels of the intermediate size or grey zone fragile X mental retardation 1 alleles are raised, and correlate with the number of CGG repeats. J Med Genet.

[125] Loesch DZ (2012). Fragile X-associated tremor/ataxia phenotype in a male carrier of unmethylated full mutation in the FMR1 gene. Clin Genet.

[126] Pretto DI (2013). Intranuclear inclusions in a fragile X mosaic male. Transl Neurodegener.

[127] Landrigan PJ (2010). What causes autism? Exploring the environmental contribution. Curr Opin Pediatr.

[128] Pessah IN, Cherednichenko G, Lein PJ (2010). Minding the calcium store: Ryanodine receptor activation as a convergent mechanism of PCB toxicity. Pharmacol Ther.

[129] Halladay AK (2009). Animal models of autism spectrum disorders: information for neurotoxicologists. Neurotoxicology.

[130] Trnovec T (2011). Assessment of exposure to PCB 153 from breast feeding and normal food intake in individual children using a system approach model. Chemosphere.

[131] Hertz-Picciotto I (2006). The CHARGE study: an epidemiologic investigation of genetic and environmental factors contributing to autism. Environ Health Perspect.

[132] Hertz-Picciotto I (2011). Polybrominated diphenyl ethers in relation to autism and developmental delay: a case-control study. Environ Health.

[133] Wolstenholme JT (2012). Gestational exposure to bisphenol a produces transgenerational changes in behaviors and gene expression. Endocrinology.

[134] Wilder RT (2009). Early exposure to anesthesia and learning disabilities in a population-based birth cohort. Anesthesiology.

[135] Hudson AE, Hemmings HC (2011). Are anaesthetics toxic to the brain?. Br J Anaesth.

[136] Tang JX (2011). Human Alzheimer and inflammation biomarkers after anesthesia and surgery. Anesthesiology.

[137] Xu B (2006). Heroin-administered mice involved in oxidative stress and exogenous antioxidantalleviated withdrawal syndrome. Basic Clin Pharmacol Toxicol.

[138] Ramirez SH (2009). Methamphetamine disrupts blood-brain barrier function by induction of oxidative stress in brain endothelial cells. J Cereb Blood Flow Metab.

[139] Muriach M (2010). Cocaine causes memory and learning impairments in rats: involvement of nuclear factor kappa B and oxidative stress, and prevention by topiramate. J Neurochem.

[140] Sarafian TA (1999). Oxidative stress produced by marijuana smoke. An adverse effect enhanced by cannabinoids. Am J Respir Cell Mol Biol.

[141] Pan J (2005). Oxidative stress in heroin administered mice and natural antioxidants protection. Life Sci.

[142] Geibprasert S, Gallucci M, Krings T (2010). Addictive illegal drugs: structural neuroimaging. AJNR Am J Neuroradiol.

[143] Zalesky A (2012). Effect of long-term cannabis use on axonal fibre connectivity. Brain.

[144] Dorn MB, Mazzocco MM, Hagerman RJ (1994). Behavioral and psychiatric disorders in adult male carriers of fragile X. J Am Acad Child Adolesc Psychiatry.

[145] Kogan CS (2008). Impact of the Fragile X mental retardation 1 (FMR1) gene premutation on neuropsychiatric functioning in adult males without fragile X-associated Tremor/Ataxia syndrome: a controlled study. Am J Med Genet B Neuropsychiatr Genet.

[146] Schumann G (2008). Systematic analysis of glutamatergic neurotransmission genes in alcohol dependence and adolescent risky drinking behavior. Arch Gen Psychiatry.

[147] Blednov YA, Harris RA (2008). Metabotropic glutamate receptor 5 (mGluR5) regulation of ethanol sedation, dependence and consumption: relationship to acamprosate actions. Int J Neuropsychopharmacol.

[148] Fukami G (2004). Effect of antioxidant N-acetyl-L-cysteine on behavioral changes and neurotoxicity in rats after administration of methamphetamine. Brain Res.

[149] Bajic D, Commons KG, Soriano SG (2013). Morphine-enhanced apoptosis in selective brain regions of neonatal rats. Int J Dev Neurosci.

[150] Kofke WA (1996). Opioid neurotoxicity: fentanyl dose-response effects in rats. Anesth Analg.

[151] Prins ND (2005). Cerebral small-vessel disease and decline in information processing speed, executive function and memory. Brain.

[152] Gouw AA (2006). Simple versus complex assessment of white matter hyperintensities in relation to physical performance and cognition: the LADIS study. J Neurol.

[153] Alfonso-Loeches S (2010). Pivotal role of TLR4 receptors in alcohol-induced neuroinflammation and brain damage. J Neurosci.

[154] Alfonso-Loeches S (2012). Toll-like receptor 4 participates in the myelin disruptions associated with chronic alcohol abuse. Glia.

[155] Bava S (2013). Longitudinal Changes in White Matter Integrity Among Adolescent Substance Users. Alcoholism-Clinical and Experimental Research.

[156] Yuan H (2013). Neuroprotective effects of resveratrol on embryonic dorsal root ganglion neurons with neurotoxicity induced by ethanol. Food Chem Toxicol.

[157] Meredith RM, Dawitz J, Kramvis I (2012). Sensitive time-windows for susceptibility in neurodevelopmental disorders. Trends Neurosci.

[158] Seritan AL (2014). Memantine for fragile X-Associated Tremor/Ataxia Syndrome: A Randomized, Double-Blind, Placebo-Controlled Trial. J Clin Psychiatry.

[159] Yang J-C Effects of Memantine on Semantic Memory in Fragile X-associated Tremor/Ataxia Syndrome (FXTAS) as Measured by Event-related Potentials.

[160] Bourgeois JA (2009). A review of fragile X premutation disorders: expanding the psychiatric perspective. J Clin Psychiatry.

[161] Bianchi P (2010). Early pharmacotherapy restores neurogenesis and cognitive performance in the Ts65Dn mouse model for Down syndrome. J Neurosci.

[162] Jacobs BL, van Praag H, Gage FH (2000). Adult brain neurogenesis and psychiatry: a novel theory of depression. Mol Psychiatry.

[163] Santarelli L (2003). Requirement of hippocampal neurogenesis for the behavioral effects of antidepressants. Science.

[164] Mostert JP (2008). Therapeutic potential of fluoxetine in neurological disorders. CNS Neurosci Ther.

[165] de Diego-Otero Y (2009). Alpha-tocopherol protects against oxidative stress in the fragile X knockout mouse: an experimental therapeutic approach for the Fmr1 deficiency. Neuropsychopharmacology.

[166] Au J, Hagerman R, Pfaff D (2013). Fragile X–Associated Disorders. Neuroscience in the 21st Century.

[167] Dysken MW (2014). Effect of vitamin E and memantine on functional decline in Alzheimer disease: the TEAM-AD VA cooperative randomized trial. JAMA.

[168] Romero-Zerbo Y (2009). Protective effects of melatonin against oxidative stress in Fmr1 knockout mice: a therapeutic research model for the fragile X syndrome. J Pineal Res.

[169] Wirojanan J (2009). The efficacy of melatonin for sleep problems in children with autism, fragile X syndrome, or autism and fragile X syndrome. J Clin Sleep Med.

[170] Chonchaiya W (2010). Clinical involvement in daughters of men with fragile X-associated tremor ataxia syndrome. Clin Genet.

[171] Srinivasan V (2011). Therapeutic potential of melatonin and its analogs in Parkinson's disease: focus on sleep and neuroprotection. Ther Adv Neurol Disord.

[172] Srinivasan V (2011). Melatonin in mitochondrial dysfunction and related disorders. Int J Alzheimers Dis.

[173] Hagerman RJ (1986). Oral folic acid versus placebo in the treatment of males with the fragile X syndrome. Am J Med Genet.

[174] Hagerman RJ, Hagerman PJ, Hagerman RJ, Hagerman PJ (2002). Fragile X Syndrome: Diagnosis, Treatment, and Research.

[175] Smith AD (2010). Homocysteine-lowering by B vitamins slows the rate of accelerated brain atrophy in mild cognitive impairment: a randomized controlled trial. PLoS One.

[176] Shults CW (2002). Effects of coenzyme Q10 in early Parkinson disease: evidence of slowing of the functional decline. Arch Neurol.

[177] Muller T (2003). Coenzyme Q10 supplementation provides mild symptomatic benefit in patients with Parkinson's disease. Neurosci Lett.

[178] Dumont M (2011). Coenzyme Q10 decreases amyloid pathology and improves behavior in a transgenic mouse model of Alzheimer's disease. J Alzheimers Dis.

[179] Cooper JM, Schapira AH (2007). Friedreich's ataxia: coenzyme Q10 and vitamin E therapy. Mitochondrion.

[180] Hickey MA (2012). Evidence for behavioral benefits of early dietary supplementation with CoEnzymeQ10 in a slowly progressing mouse model of Huntington's disease. Mol Cell Neurosci.

[181] Dong GZ (2013). Red ginseng abrogates oxidative stress via mitochondria protection mediated by LKB1-AMPK pathway. BMC Complement Altern Med.

[182] Park JS (2009). Anti-inflammatory mechanism of ginseng saponins in activated microglia. J Neuroimmunol.

[183] Dang H (2009). Antidepressant effects of ginseng total saponins in the forced swimming test and chronic mild stress models of depression. Prog Neuropsychopharmacol Biol Psychiatry.

[184] Zhao H (2009). Long-term ginsenoside administration prevents memory impairment in aged C57BL/6J mice by up-regulating the synaptic plasticity-related proteins in hippocampus. Behav Brain Res.

[185] Tayyebi-Khosroshahi H (2010). Effect of omega-3 fatty acid on oxidative stress in patients on hemodialysis. Iran J Kidney Dis.

[186] Garrel C (2012). Omega-3 fatty acids enhance mitochondrial superoxide dismutase activity in rat organs during post-natal development. Int J Biochem Cell Biol.

[187] Zararsiz I (2011). Protective effects of omega-3 essential fatty acids against formaldehyde-induced cerebellar damage in rats. Toxicol Ind Health.

[188] Schroeder EK (2009). Green tea epigallocatechin 3-gallate accumulates in mitochondria and displays a selective antiapoptotic effect against inducers of mitochondrial oxidative stress in neurons. Antioxid Redox Signal.

[189] Andrade JP, Assuncao M (2012). Protective effects of chronic green tea consumption on age-related neurodegeneration. Curr Pharm Des.

[190] Panickar KS, Polansky MM, Anderson RA (2009). Green tea polyphenols attenuate glial swelling and mitochondrial dysfunction following oxygen-glucose deprivation in cultures. Nutr Neurosci.

[191] Itoh T (2011). (−)-Epigallocatechin-3-gallate protects against neuronal cell death and improves cerebral function after traumatic brain injury in rats. Neuromolecular Med.

[192] Kelsey N (2011). Neuroprotective effects of anthocyanins on apoptosis induced by mitochondrial oxidative stress. Nutr Neurosci.

[193] Mischley LK, Allen J, Bradley R (2012). Coenzyme Q10 deficiency in patients with Parkinson's disease. J Neurol Sci.

[194] Shults CW, Haas R (2005). Clinical trials of coenzyme Q10 in neurological disorders. Biofactors.

[195] Huang CC (2009). Protective effects of L-arginine supplementation against exhaustive exerciseinduced oxidative stress in young rat tissues. Chin J Physiol.

[196] Mandel SA (2012). Molecular mechanisms of the neuroprotective/neurorescue action of multitarget green tea polyphenols. Front Biosci (Schol Ed).

[197] Bowman GL (2012). Nutrient biomarker patterns, cognitive function, and MRI measures of brain aging. Neurology.

[198] Gane L, Cronister A, Hagerman RJ, Hagerman PJ (2002). Genetic Counseling, in The Fragile X Syndrome: Diagnosis, Treatment, and Research.

[199] Brouwer JR (2008). Altered hypothalamus-pituitary-adrenal gland axis regulation in the expanded CGG-repeat mouse model for fragile X-associated tremor/ataxia syndrome. Psychoneuroendocrinology.

[200] Foley E (2010). Mindfulness-based cognitive therapy for individuals whose lives have been affected by cancer: a randomized controlled trial. J Consult Clin Psychol.

[201] Grossman P (2010). MS quality of life, depression, and fatigue improve after mindfulness training: a randomized trial. Neurology.

[202] Sephton SE (2007). Mindfulness meditation alleviates depressive symptoms in women with fibromyalgia: results of a randomized clinical trial. Arthritis Rheum.

[203] Pradhan EK (2007). Effect of Mindfulness-Based Stress Reduction in rheumatoid arthritis patients. Arthritis Rheum.

[204] Leehey MA (2011). Fibromyalgia in fragile X mental retardation 1 gene premutation carriers. Rheumatology (Oxford).

[205] Grossman P (2004). Mindfulness-based stress reduction and health benefits. A meta-analysis. J Psychosom Res.

[206] Maime M Relaxation Techniques for Health: An Introduction.

[207] Lubar JF, Shouse MN (1976). EEG and behavioral changes in a hyperkinetic child concurrent with training of the sensorimotor rhythm (SMR): a preliminary report. Biofeedback Self Regul.

[208] Thompson L, Thompson M, Reid A (2010). Neurofeedback outcomes in clients with Asperger's syndrome. Appl Psychophysiol Biofeedback.

[209] Prinsloo GE (2011). The effect of short duration heart rate variability (HRV) biofeedback on cognitive performance during laboratory induced cognitive stress. Applied Cognitive Psychology.

[210] Lehrer PM, Vaschillo E, Vaschillo B (2000). Resonant frequency biofeedback training to increase cardiac variability: rationale and manual for training. Appl Psychophysiol Biofeedback.

[211] Lloyd A, Brett D, Wesnes K (2010). Coherence training in children with attention-deficit hyperactivity disorder: cognitive functions and behavioral changes. Altern Ther Health Med.

[212] Bradley RT (2010). Emotion self-regulation, psychophysiological coherence, and test anxiety: results from an experiment using electrophysiological measures. Appl Psychophysiol Biofeedback.

[213] Pierce GL (2011). Habitually exercising older men do not demonstrate age-associated vascular endothelial oxidative stress. Aging Cell.

[214] Shanely RA (2013). Inflammation and oxidative stress are lower in physically fit and active adults. Scand J Med Sci Sports.

[215] Hagerman R (2012). Fragile X syndrome and targeted treatment trials. Results Probl Cell Differ.

[216] Traustadottir T, Bosch PR, Matt KS (2005). The HPA axis response to stress in women: effects of aging and fitness. Psychoneuroendocrinology.

[217] Wolf SA, Melnik A, Kempermann G (2011). Physical exercise increases adult neurogenesis and telomerase activity, and improves behavioral deficits in a mouse model of schizophrenia. Brain Behav Immun.

[218] Speisman RB (2013). Daily exercise improves memory, stimulates hippocampal neurogenesis and modulates immune and neuroimmune cytokines in aging rats. Brain Behav Immun.

[219] Jenkins EC (2012). Reduced telomere length in individuals with FMR1 premutations and full mutations. Am J Med Genet A.

[220] Erickson KI (2011). Exercise training increases size of hippocampus and improves memory. Proc Natl Acad Sci U S A.

[221] Ferreira AF (2011). Short-term, moderate exercise is capable of inducing structural, BDNF-independent hippocampal plasticity. Brain Res.

[222] Eyre H, Baune BT (2012). Neuroimmunological effects of physical exercise in depression. Brain Behav Immun.

[223] Suijo K (2013). Resistance exercise enhances cognitive function in mouse. Int J Sports Med.

[224] Tarnopolsky MA (2009). Mitochondrial DNA shifting in older adults following resistance exercise training. Appl Physiol Nutr Metab.

[225] Little JP (2010). A practical model of low-volume high-intensity interval training induces mitochondrial biogenesis in human skeletal muscle: potential mechanisms. J Physiol.

[226] Willis SL (2006). Long-term effects of cognitive training on everyday functional outcomes in older adults. JAMA.

[227] Jaeggi SM (2011). Short- and long-term benefits of cognitive training. Proc Natl Acad Sci U S A.

